# Cacao consumption improves passive avoidance memory impairment in a rat model of Alzheimer’s disease: the role of hippocampal synaptic plasticity and oxidative stress

**DOI:** 10.3389/fphar.2024.1379264

**Published:** 2024-05-02

**Authors:** Hamid Shokati Basir, Naser Mirazi, Alireza Komaki, Abdolkarim Hosseini

**Affiliations:** ^1^ Department of Biology, Faculty of Basic Science, Bu-Ali Sina University, Hamedan, Iran; ^2^ Neurophysiology Research Center, Hamadan University of Medical Sciences, Hamadan, Iran; ^3^ Faculty of Life Sciences and Biotechnology, Shahid Beheshti University, Tehran, Iran

**Keywords:** Alzheimer’s disease, cacao, passive avoidance memory, long-term potentiation, oxidative stress

## Abstract

**Introduction:** Alzheimer’s disease (AD) causes progressive loss of cognitive function and synaptic plasticity, which is the most common form of dementia. The present study was designed to scrutinize the effects of cacao on passive avoidance memory function and to identify the roles of hippocampal synaptic plasticity and oxidative stress in an AD rat model induced by unilateral intracerebroventricular (UICV) injection of amyloid-beta (Aβ).

**Methods:** Oral administration of cacao (500 mg/kg/ day) was given for 2 consecutive months. A memory retention test was conducted 24 h after passive avoidance training was completed. Subsequently, the amplitude of population spike (PS) and slope of field excitatory postsynaptic potentials (fEPSPs) were assessed at hippocampal long-term potentiation (LTP) in perforant pathway–dentate gyrus (PP-DG) synapses. Moreover, total thiol group (TTG) and malondialdehyde (MDA) concentrations were evaluated in the plasma. Furthermore, compact Aβ plaques were detected in the hippocampal DG by performing Congo red staining.

**Results:** As a result of AD induction, passive avoidance memory was impaired; also, reduced fEPSP slopes, PS amplitudes, and content of TTG, and increase in MDA levels in the rats were observed. In contrast, cacao treatment ameliorated passive avoidance memory impairment, improved hippocampal LTP impairment, modulated oxidative–antioxidative status, and delayed Aβ plaques production in AD rats.

**Disscussion:** Conclusively, cacao alleviates Aβ-induced cognitive deficit, probably by the amelioration of hippocampal LTP impairment, modulation of oxidative–antioxidative status, and inhibition of Aβ plaque accumulation

## 1 Introduction

The most common neurodegenerative disorder, namely, Alzheimer’s disease (AD), affects learning and memory processes (De Strooper and Karran, 2016). It causes amyloid-beta (Aβ) plaque formation and synaptic transmission impairment (Srivastava et al., 2021). This neuropathological condition of AD in the cellular phase is depicted parallel with widespread Aβ deposition as extracellular neurotic plaques. Aβ induces the spread of neurofibrillary tangle (NFT) formation in the brain, which contains hyperphosphorylated tau protein. These factors lead to dysfunction of synapses and extensive neurodegeneration in the basal forebrain cholinergic neurons (Long and Holtzman, 2019; Ma et al., 2021). Aβ is produced by β- and γ-secretases by sequential proteolytic cleavage of amyloid precursor protein (APP) (Zhang et al., 2018). Aβ plays a causal role in mitochondrial dysfunction, disrupting the equilibrium between oxidants and antioxidants and raising oxidative stress in the body (Chauhan and Chauhan, 2006). The reduction of antioxidants or accumulation of free radicals like reactive oxygen species (ROS) in cells during oxidative stress ultimately leads to the damage of cellular functions and cell death in the central nervous system of affected individuals (Birben et al., 2012). Although none of the existing models of AD completely replicate the human disease, Aβ-induced AD is used as a well-defined model to identify the underlying pathophysiological mechanisms of AD (Arabi et al., 2023; Bagheri et al., 2023). Previous research workers have highlighted that Aβ peptides result in disrupted synaptic plasticity in the hippocampus and impairment of learning and memory, and as a result, there is cognitive decline (Ahmadi et al., 2021b).

A wide range of compounds, such as dietary polyphenols, mainly isolated from plants have beneficial effects for the treatment and prevention of AD. Natural compounds function by a variety of therapeutic mechanisms such as preventing Aβ aggregation, promotion of Aβ clearance, oxidative stress control via ROS scavenging, and Aβ-induced inflammatory response (Andrade et al., 2019). Cacao, a food of plant origin (*Theobroma cacao*), is a major source of varied polyphenol contents such as quercetin, clovamide, procyanidin, epicatechin, and catechin, and also methylxanthines such as theobromine and caffeine, and has different levels of antioxidant capacity (Cronquist, 1981; Arlorio et al., 2008; Noori et al., 2009). Research studies demonstrate that cacao, with its potent antioxidant activity, avoided neuroinflammation, neurodegeneration, and cognitive decline affecting cognitive functions (Sokolov et al., 2013; Zeli et al., 2022). Therefore, considerable attention has been paid to the potential benefits of cacao aimed at limiting the progressive loss of cognitive processes.

In light of this background, this study aimed to indicate how oral cacao powder administration affected passive avoidance memory function, long-term potentiation (LTP) induction in the hippocampal dentate gyrus (DG), oxidative stress biomarkers, and Aβ plaque development in hippocampal DG in male rats after unilateral intracerebroventricular (UICV) administration of Aβ.

## 2 Materials and methods

### 2.1 Animals and experimental design

Male Wistar rats with a weight of 200–220 g were attained from Hamadan University of Medical Sciences animal house (Hamadan, Iran). Three rats were housed in each standard laboratory cage with *ad libitum* access to rodent pellets (47% carbohydrate, 5% fat, 23% protein, 5% fiber, 20% water, vitamins, and minerals) and tap water. The animal room had a controlled temperature of 22°C ± 2°C with 60% ± 5% comparative humidity and alternated 12-h light–dark cycle. Experimental methods and animal care were in accordance with the National Institutes of Health (NIH) and ARRIVE Guidelines and were approved by Bu-Ali Sina University-Hamedan’s Ethics Committee (Ethic code: IR.BASU.REC.1398.025).

As described below, the rats were separated into seven groups of eight rats following 1 week of adaptation:

I: Control group: rats received 5 mL/kg/day of 0.9% normal saline via oral gavage (P.O.).
II: Sham group: rats received a stereotaxic UICV injection of phosphate-buffered saline (PBS) (5 μL/rat; 10 mmol/L).
III: Cacao *per se* group: rats received cacao (500 mg/kg/day; P.O. for 60 days).
IV: Pre-Aβ group: rats received a stereotaxic UICV injection of Aβ (5 μg/5 μL/rat) on the 67th day.
V: Pre-treat group: rats received cacao (500 mg/kg/day; P.O. for 60 days) before a stereotaxic UICV injection of Aβ (5 μg/5 μL/rat).
VI: Post-Aβ group: rats received a stereotaxic UICV injection of Aβ (5 μg/5 μL/rat) on the 7th day.
VII: Post-treat group: rats received cacao (500 mg/kg/day; P.O. for 60 days) after the stereotaxic UICV injection of Aβ (5 μg/5 μL/rat).

**FIGURE 1:**
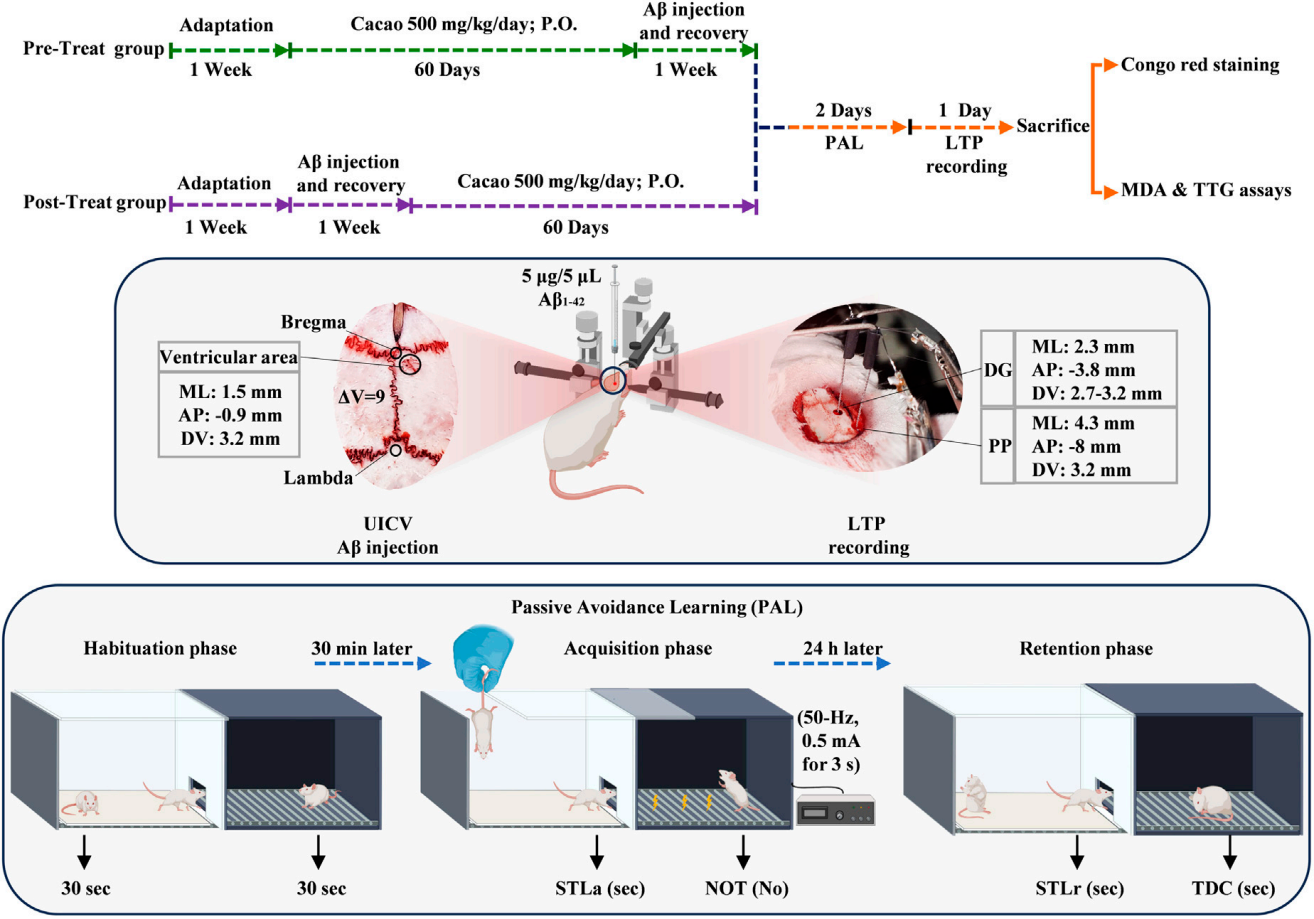
Following 1 week of adaptation, prior to (pre-treat group) and after (post-treat group) Aβ solution (5 μg/5 μL/rat) unilateral intracerebroventricular (UICV) injections at a rate of 1 μL/min; cacao (500 mg/kg bw daily) was administered by oral gavage for 60 days. Subsequently, passive avoidance learning and LTP recording were performed. At the end of the experiments, the levels of biomarkers (MDA, TTG) were determined by plasma assays, and Congo red staining was performed on the hippocampal tissue.

Cacao (415 kcal/100 g, fat, protein, carbohydrate, fiber, and salt; Cadbury Co., UK) was prepared immediately before use and administered once a day for 60 consecutive days by oral gavage. In previous studies, cacao and cacao products have been administrated for durations ranging from 21 days (Noori et al., 2009) to 3 months (Madhavadas et al., 2016) and up to 1 year (Bisson et al., 2008). The dose of cacao powder was chosen according to previously published data (Noori et al., 2009; Yamada et al., 2009). Figure 1 outlines the experimental timeline.

### 2.2 AD induction

Aβ solution (2.26 × 10^−4^ mol/L) was administered to induce AD in rats. In addition, 100 μg of Aβ peptide_1–42_ rat (product No/SKU SCP0038-1 MG, Sigma Aldrich, United States) was dissolved in 100 μL of PBS. Prior to UICV injection, the Aβ was incubated at 37°C for 4 days. Amyloid fibrils are produced during this process, which are neurotoxic (Lorenzo and Yankner, 1994; Komaki et al., 2019).

For AD induction, each rat was anesthetized by intraperitoneally (I.P.) injecting a mixture of ketamine (100 mg/kg) and xylazine (10 mg/kg), and stereotaxic surgery (Dual Lab Standard Stereotaxic apparatus; Stoelting Co., Wood Dale, IL, United States) was performed, the head was shaved, and a midline sagittal incision was made in the scalp. A tiny hole was drilled carefully up to the level of the dura mater in the skull over the ventricular area (coordinates relative to bregma: medial–lateral (M/L): 1.5 mm and anterior–posterior (A/P): −0.9 mm). Hamilton syringe needle was slowly directed down to beneath the surface of the cortex for the UICV injections, into the right lateral ventricle (coordinates relative to the skull: dorsal-ventral (D/V): 3.2 mm) (Paxinos and Watson, 2006). Five μL Aβ solution was administered for 5 min (1 μL/min). The Sham group received 5 μL of PBS, which is the same as the Aβ injection. After surgery, the rats were individually placed in their cages, and with special care, they were allowed to undergo a 7-day recovery period.

### 2.3 Passive avoidance learning (PAL)

The shuttle box (Tajhiz Gostar Co, Tehran, Iran) is used to investigate the passive avoidance task as an indicator of animal learning and memory (Keikhaei et al., 2020). The apparatus is composed of a box with distinct light and dark compartments. Transparent plastic is used for the light compartment and opaque plastic is used for the dark compartment (each dimension 30 [L] × 23 [W] × 23 [H] cm), and the box is connected by a sliding door (8 × 8 cm). Electrical shock could be transmitted to the parallel stainless steel rods embedded in the floor of the dark compartment by a stimulator (Tajhiz Gostar Co, Tehran, Iran).

The rats were introduced to the lit compartment, and the sliding door was raised 30 s later to habituate them. When the rat entered the dark compartment, the door was lowered, and after 30 s, the rat was removed and moved into its cage. This test was repeated after 30 min. The first acquisition step was performed after a 30-min interval. Rats were placed in lit compartments, and a sliding door was raised 10 s later. Upon entering the dark compartment, the sliding door was shut and an electric shock (50 Hz, 0.5 mA for 3 s) was administered. Rats were returned to cages after 30 s. Two minutes later, the experiment was repeated. A step-through latency in the acquisition phase (STLa) was determined when the animal placed all four paws inside the dark compartment. The acquisition phase was terminated when the rats remained in the lit compartment for 120 consecutive seconds. The number of trials (NOT) to acquisition was recorded as an indication of the passive avoidance learning process.

After acquisition trials were completed, the retention phase was tested 24 h later. For up to 300 s, the rat was placed in the lit compartment and the sliding door was raised 5 s later. The step-through latency in the retention phase (STLr) was recorded, as was the time spent in the dark compartment (TDC). If the rat did not enter the dark compartment, a maximum score of 300 s was assigned.

### 2.4 Long-term potentiation (LTP)

The rat’s head was secured in a stereotaxic apparatus after deep anesthesia was administered with urethane (1.5 g/kg, I.P.). The locations of DG (coordinates were AP: −3.8 mm and ML: 2.3 mm relative to bregma; DV: 2.7–3.2 mm relative to the surface of the skull) and perforant pathway (PP coordinates were AP: −8 mm and ML: 4.3 mm relative to bregma; DV: 3.2 mm relative to the surface of the skull) were determined using the Paxinos atlas, and two holes were drilled in the designated points on the skull. The stimulating and recording electrodes (stainless steel with Teflon cover, 125 μm bare diameter, 175 μm coated diameter, A.M. Systems Inc., United States) were moved gently to the perforant pathway (PP) and DG, respectively (Figure 1).

**FIGURE 2:**
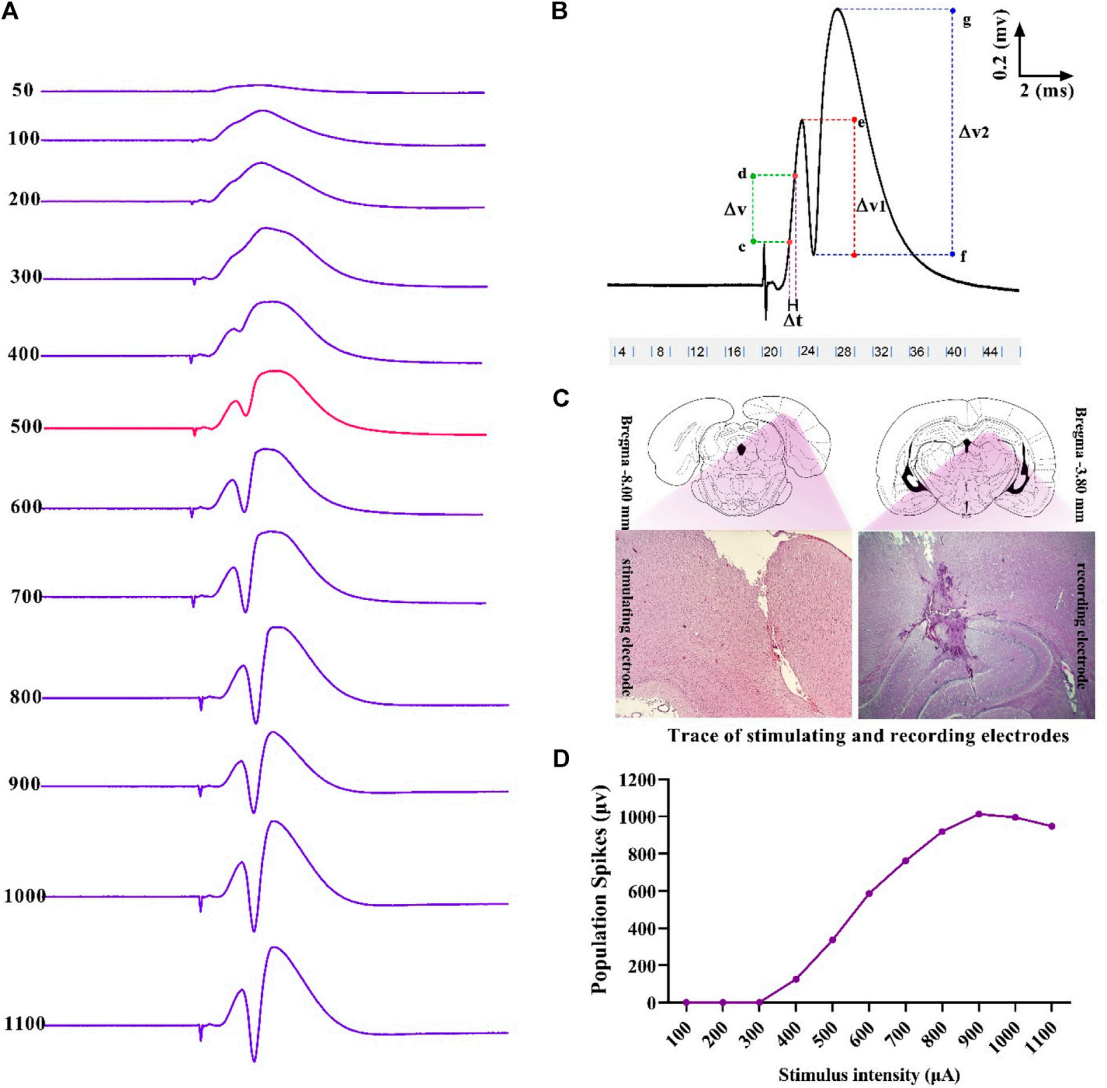
Sample of an I/O curve of PS amplitude in hippocampal DG following PP stimulation **(A)**. A representative sample of I/O trace response **(B)**. Positions and trace of stimulating and recording electrodes on the DG and PP are represented in the transverse section of the hippocampus **(C)**. Measurement of evoked potentials. Eqs 1, 2 were applied to calculate PS amplitude and fEPSP slope, respectively (see text) **(D)**. ΔV, potential difference; ΔT, time difference.

Single 0.1 ms biphasic square wave pulses at the frequency of 0.1 Hz were used for stimulation (eProbe software protocol: delay: 20,000 µs; pulse duration: 200 µs; pulse cycle: 100 µs, train: 1; trial numbers: 10; trial period: 10 s). The baseline stimulation intensity for each rat was calculated based on the input–output (I/O) curve. This curve was plotted by recording the population spike (PS) amplitude at varying intensities, and 40% of the maximum response was considered as the baseline stimulation intensity (Figure 2). When the response was stable in a 10- to 20-min control period, LTP was induced using a high-frequency stimulation (HFS) protocol of 400 Hz (10 bursts of 20 stimuli, 0.2 ms stimulus duration, and 10 s interburst interval).

Using the eLab system (ScienceBeam, Iran) and related computer software (eProbe), the PS amplitude and the slope of field excitatory postsynaptic potentials (fEPSP) were recorded at 5, 30, and 60 min after HFS in the granular cells layer of hippocampal DG following stimulation of the PP. Changes in PS amplitude and fEPSP slope were calculated during electrophysiological recordings, according to Eqs 1, 2, respectively (Figure 2):

$$\text{PS amplitude}=\frac{\Delta V1+\Delta V2}{2},$$ (1)

$$\text{fEPSP slope}=\frac{\Delta V}{\Delta T},$$ (2)

where ΔV_1_ is the potential difference between two points, with e as the peak of the first positive wave and f as the peak of the first negative deflection; ΔV_2_ is the potential difference between two points, with g as the peak of the second positive wave and f as the peak of the first negative deflection; ΔT is the time difference between two points c and d; and ΔV is the potential difference between two points c and d that were between 20% and 80% of the first positive wave.

The values of the fEPSP slope and the PS amplitude at 5, 30, and 60 min were normalized relative to their baselines to measure the LTP magnitude (Eq. 3). Significant increase (*p* < 0.05) in PS amplitude and fEPSP slope (% change) was considered as a successful induction of LTP.

$$\text{LTP}=\frac{\text{PS}\;\text{amplitude}\;\text{or}\;\text{fEPSP}\;\text{slope}\;\text{after}\;\text{HFS}\times 100}{\text{PS}\;\text{amplitude}\;\text{or}\;\text{fEPSP}\;\text{slope}\;\text{at}\;\text{baseline}}.$$ (3)

### 2.5 Biochemical assay

After LTP recording, the intra-cardiac blood samples were collected in heparinized tubes. Following centrifugation at 3,500 rpm for 20 min, clear plasma was separated into 100 µL aliquots and stored at −20°C.

Rat malondialdehyde (MDA) and total thiol group (TTG) assay kits (Kiazist Life Sciences, Iran) were used to calculate the values of oxidant and antioxidant biomarkers, respectively, based on the manufacturer’s procedures.

### 2.6 Histology analysis

Animals were perfused transcardially using ice-cold saline with a 10% formalin solution. A 10% formalin solution was applied to the harvested brains and left for 72 h before they were embedded in paraffin. The sections were cut into 5-mm-thick halves. After deparaffinization and rehydration, sections were washed in distilled water. Congo red staining was performed according to standard procedures for 5 min. In the next step, slides were washed in distilled water and differentiated in an alcoholic potassium hydroxide (KOH) solution. The slides were then counterstained with hematoxylin (Merck Co., Germany) for 3 minutes, dehydrated in graded alcohol and xylene, and mounted. The hippocampal DG region of each section was examined for Aβ plaque formation. The amyloid deposits were stained red. Two fields of view from each slide were captured at magnification 400× under a light microscope (Olympus PX 50 F3 model, Japan).

### 2.7 Statistical analysis

Data were analyzed and plotted using GraphPad Prism software, version 9.0 (GraphPad Software, San Diego, CA, United States). The Shapiro–Wilk test was used to check that the data were normally distributed before performing a one-way or two-way analysis of variance (ANOVA). As part of the PAL, NOT, STLr, and TDC parameters were analyzed using Kruskal–Wallis (ANOVA on ranks) and Dunn’s test for multiple comparisons, and data were represented using box and whisker plots, displaying medians, interquartile ranges, maximums, and minimums. In LTP, repeated-measures two-way ANOVA (two-way RM ANOVA) was used to compare fEPSP slope and PS amplitude trends. Other data were subjected to a parametric ANOVA followed by a Tukey’s *post hoc* test; the results are displayed as mean ± standard deviation (mean ± SD). Statistical significance was defined as *p* values below 0.05 in all analyses.

## 3 Results

### 3.1 Body weight

**FIGURE 3:**
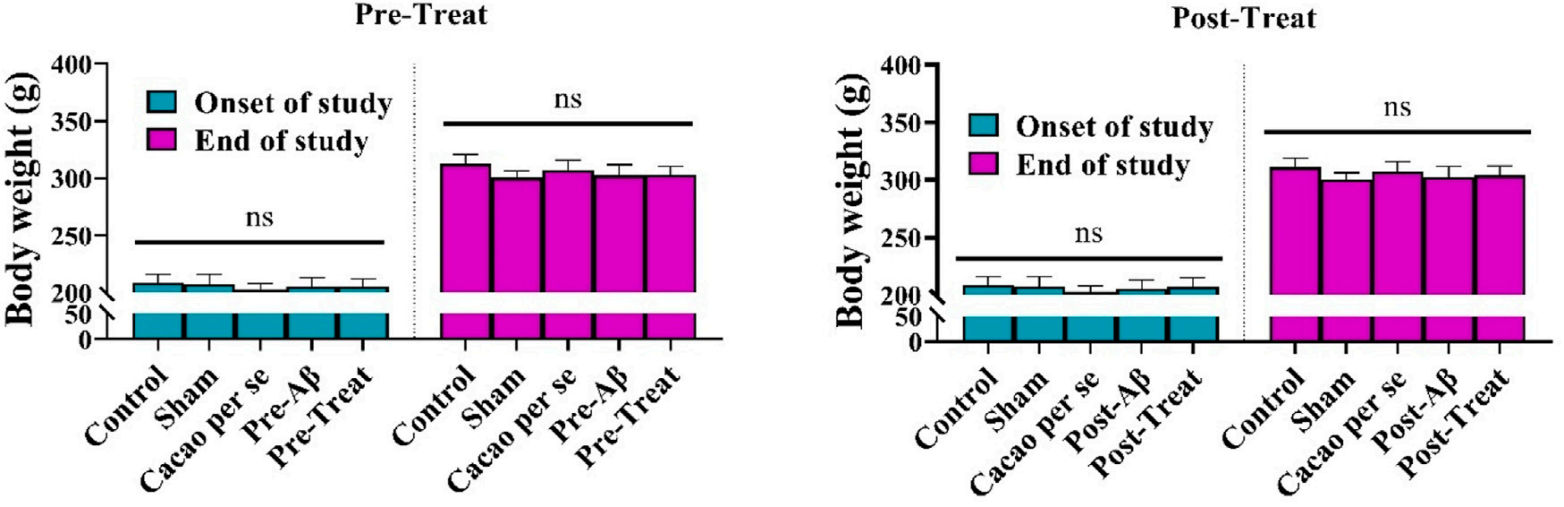
Effects of cacao (500 mg/kg/day, for 60 consecutive days) on body weights of AD rats. Data are presented as means ± SD of eight animals per group (one-way ANOVA). ns, no significance.

There was no significant difference in body weight between pre-treat groups at the beginning of the study (F _(4, 35)_ = 0.74, *p* = 0.568) and the end of the study (F _(4, 35)_ = 3.012; *p* = 0.030, Figure 3). Furthermore, the body weight between post-treat groups did not differ significantly at the beginning of the study (F _(4, 35)_ = 0.723, *p* = 0.581) and at the end of the study (F _(4, 35)_ = 2.04; *p* = 0.110, Figure 3).

### 3.2 The effects of cacao on the PAL in different groups

**FIGURE 4:**
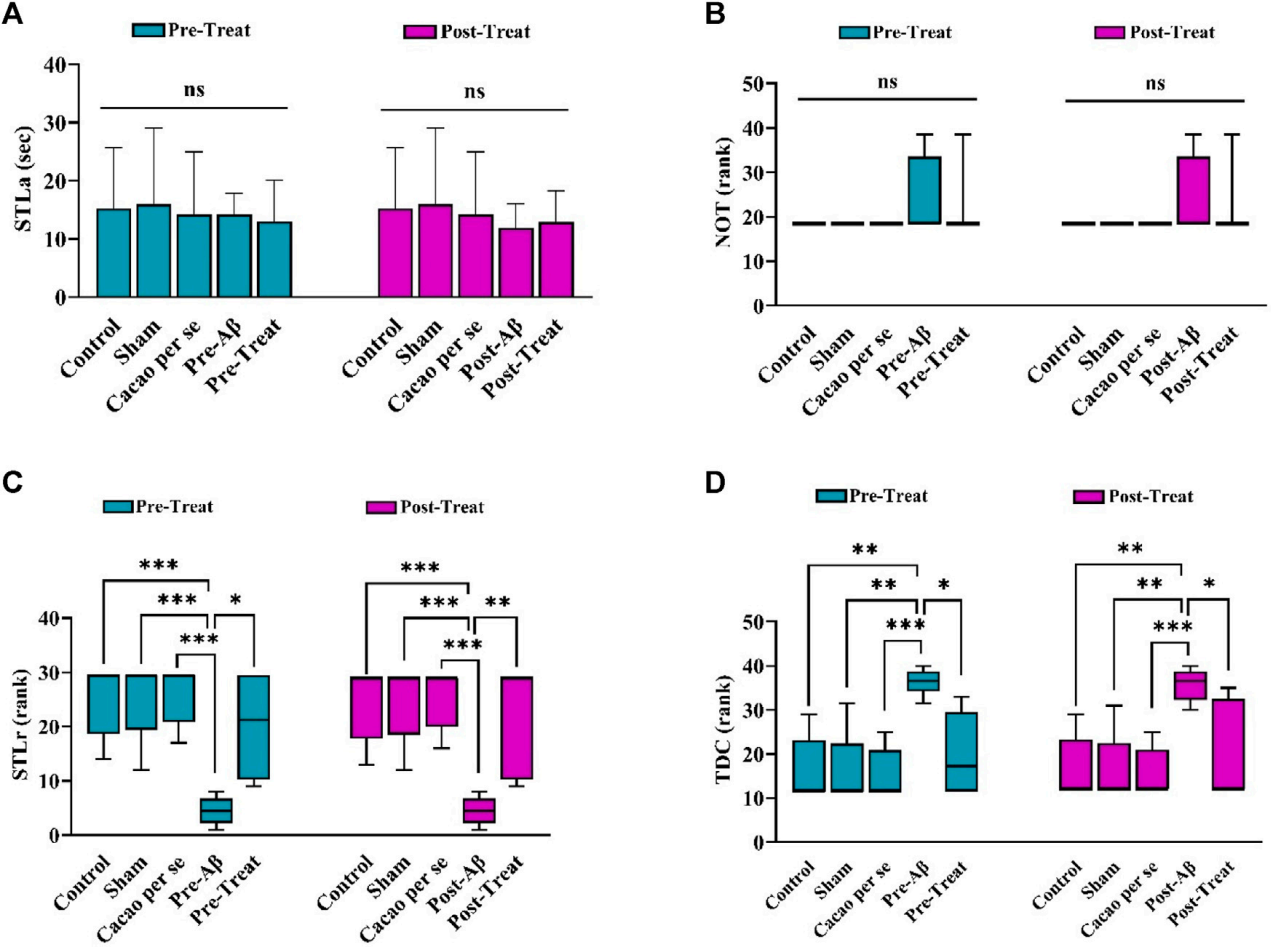
Effects of cacao supplementation (500 mg/kg/day, for two consecutive months) on PAL in the Aβ rats (*n* = 8). **(A)** Step-through latency in the acquisition trial (STLa) and is presented as means ± SD (one-way ANOVA). **(B–D)** The number of trials to reach learning (NOT), step-through latency in the retention phase (STLr), and time spent in the dark compartment (TDC), respectively. Data are presented as the median interquartile range (Kruskal Wallis test and Dunn’s *post hoc* test). ns, no significance; *p < 0.05, **p < 0.01, and ***p < 0.001.

A comparison of the STLa among the pre-treat (F _(4, 35)_ = 0.113; *p* = 0.98, Figure 4A) and post-treat (F _(4, 35)_ = 0.258; *p* = 0.902, Figure 4A) groups showed no significant differences in the performance of the rats in the acquisition phase of PAL. Furthermore, the experimental groups did not differ significantly in the term of NOT factor in pre-treat (H _(4)_ = 5.62; *p* = 0.23, Figure 4B) and post-treat groups (H _(5)_ = 5.62; *p* = 0.229, Figure 4B).

In addition, STLr in the pre-Aβ group (H _(4)_ = 24.3, *p* < 0.001, Figure 4C) and post-Aβ groups (H _(4)_ = 23.81, *p* < 0.001, Figure 4C) was significantly lower than that in the control group in the retention phase (*p* < 0.001).

Significant differences were also observed in TDC between the pre-Aβ (H _(4)_ = 23.1, *p* < 0.001, Figure 4D) and post-Aβ (H _(4)_ = 21.52, *p* < 0.001, Figure 4D) groups, so the Aβ rats spent more time in the dark compartment than the control group (*p* = 0.001 for each comparison). Cacao consumption in the Aβ rats prevented these changes in the pre-treat (*p* = 0.02) and post-treat (*p* = 0.023) groups in comparison to the Aβ rats.

### 3.3 The effects of cacao on the fEPSP slope and PS amplitude of DG granular cell layer in different groups

**FIGURE 5:**
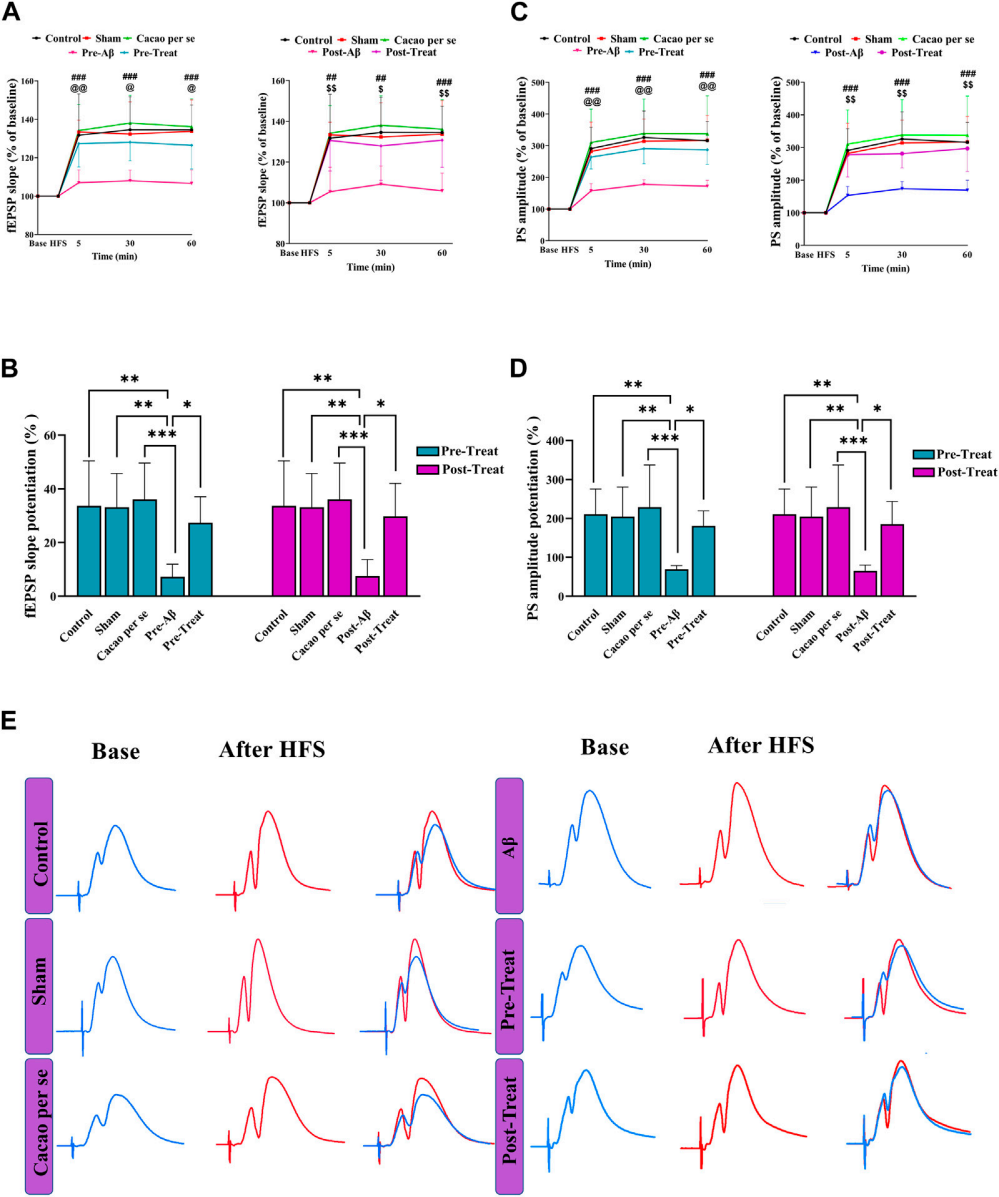
Effects of cacao supplementation (500 mg/kg/day for 60 consecutive days) on the evoked field potential in the hippocampal dentate gyrus (DG) after high-frequency stimulation (HFS) in AD rats. **(A,C)** Time course diagrams showing the changes in fEPSP slope and PS amplitude, respectively, prior to HFS and 5, 30, and 60 min after HFS of the PP (two-way RM ANOVA and Tukey’s *post hoc* test). ##*p* < 0.01, ###*p* < 0.001 compared between control and pre-Aβ groups or control and post-Aβ groups; @ *p* < 0.05, @@ *p* < 0.01 compared between pre-Aβ and pre-treat groups; $ *p* < 0.05, $$ *p* < 0.01 compared between post-Aβ and post-treat groups. **(B,D)** Percentage changes of fEPSP slope and PS amplitude during LTP induction, respectively (one-way ANOVA and Tukey’s *post hoc* test). Data are expressed as means ± SD % of baseline of eight animals per group. *p < 0.05, **p < 0.01, and ***p < 0.001. **(E)** Evoked field potentials in the DG of the experimental groups were measured before and 30 min after HFS.

Field potentials were recorded from the hippocampal DG after stimulation of the hippocampal PP. According to Figures 5A, E, sample of the evoked field potential in the DG was recorded before HFS delivery (stable baseline response) and after tetanus.

DG’s fEPSP slope in the pre-treat group was significantly affected by both time points (F _(3, 28)_ = 101.7, *p* < 0.001) and treatment (F _(4, 112)_ = 17.05, *p* < 0.001) in a two-way RM ANOVA (Figure 5A). Tukey’s *post hoc* analysis indicated significant decrease at different time periods: 5 (*p* < 0.001), 30 (*p* < 0.001), and 60 min (*p* < 0.001) after HFS in the pre-Aβ group compared with the control group. The supplementation of cacao (500 mg/kg/day for 60 consecutive days) prevented the decremental effect of Aβ on the slope of fEPSP in the pre-treat group at different time periods: 5 (*p* = 0.009), 30 (*p* = 0.010), and 60 min (*p* < 0.011); so, their magnitudes were similar to that of animals in the control group.

fEPSP slope in the post-treat group was significantly affected by both time points (F _(3, 28)_ = 100.3, *p* < 0.001) and treatment (F _(4, 112)_ = 14.79, *p* < 0.001) in a two-way RM ANOVA (Figure 5A). Tukey’s *post hoc* analysis indicated significant decrease at different time periods: 5 (*p* < 0.001), 30 (*p* = 0.001), and 60 min (*p* < 0.001) after HFS in the post-Aβ group compared with the control group. The supplementation of cacao (500 mg/kg/day for 60 consecutive days) ameliorated the decremental effect of Aβ on the slope of fEPSP in the post-treat group at different time periods: 5 (*p* = 0.002), 30 (*p* = 0.042), and 60 min (*p* < 0.002).

One-way analysis of variance showed a significant difference in the mean percent fEPSP slope change during 60 min after HFS between the pre-treat (F _(4, 35)_ = 6.71, *p* = 0.0004) and post-treat groups (F _(4, 35)_ = 7.48, *p* = 0.0002). According to the *post hoc* Tukey’s test, there was a significant decrease in the pre-Aβ (*p* = 0.001) and post-Aβ (*p* = 0.002) groups compared to the control group (Figure 5B). In addition, it was significantly increased after cacao treatment in pre-treat group in comparison to the pre-Aβ group (*p* = 0.017, Figure 5B), and in the post-treat group in comparison to the post-Aβ group (*p* = 0.010, Figure 5B). This suggests that HFS did not considerably change the fEPSP slope and LTP induction was impaired in Aβ rats.

According to the two-way RM ANOVA, the PS amplitudes of the granular cell layer are significantly influenced by time points (F _(3, 28)_ = 57.48, *p* < 0.001) and treatments (F _(4, 112)_ = 20.72, *p* < 0.001) between the pre-treat groups (Figure 5C). Tukey’s *post hoc* analysis indicated a significant decrease at different time periods: 5, 30, and 60 min after HFS in the pre-Aβ group compared with the control group (*p* < 0.001). The supplementation of cacao prevented these changes, so there was a significant difference in PS amplitudes in the pre-treat group compared to the pre-Aβ group at different time periods: 5 (*p* = 0.004), 30 (*p* = 0.002), and 60 min (*p* < 0.001) (Figure 5C).

PS amplitudes of the granular cell layer in the post-treat group was significantly affected by both time points (F _(3, 28)_ = 63.42, *p* < 0.001) and treatment (F _(4, 112)_ = 18.87, *p* < 0.001) in a two-way RM ANOVA (Figure 5C). Tukey’s *post hoc* analysis indicated significant decrease at different time periods: 5 (*p* < 0.001), 30 (*p* = 0.001), and 60 min (*p* < 0.001) after HFS in the post-Aβ group compared with the control group. The supplementation of cacao (500 mg/kg/day for 60 consecutive days) ameliorated the decremental effect of Aβ on the PS amplitude in the post-treat group at different time periods: 5 (*p* = 0.001), 30 (*p* = 0.009), and 60 min (*p* < 0.001).

One-way analysis of variance showed a significant difference in the mean percent PS amplitude change during 60 min after HFS among different groups in the pre-treat (F _(4, 35)_ = 6.93, *p* = 0.0003) and post-treat (F _(4, 35)_ = 6.75, *p* = 0.0004). According to the *post hoc* Tukey’s test, there was a significant decrease in the Pre-Aβ (*p* = 0.001) and Post-Aβ (*p* = 0.002) groups compared to the control group (Figure 5D). In addition, it was significantly increased after cacao treatment in the pre-treat group in comparison to the pre-Aβ group (*p* = 0.019, Figure 5D), and the post-treat group in comparison to the post-Aβ group (*p* = 0.014, Figure 5B). This suggests that HFS did not considerably change the PS amplitude and LTP induction was impaired in the Aβ rats.

### 3.4 Effect of cacao and Aβ on TTG and MDA

**FIGURE 6:**
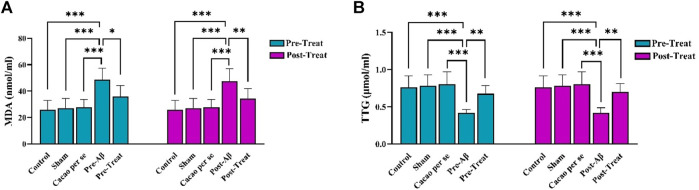
Effects of cacao supplementation (500 mg/kg/day, for two consecutive months) on the plasma parameters of malondialdehyde (MDA) **(A)** and total thiol group (TTG) **(B)** of AD rats using assay kits. Data are presented as means ± SD of eight animals per group (one-way ANOVA and Tukey’s *post hoc* test). *p < 0.05, **p < 0.01, and ***p < 0.001.

Significant differences were observed in MDA concentration among different groups in pre-treat (F _(4, 35)_ = 13.1, *p* < 0.001) and post-treat (F _(4, 35)_ = 11.51, *p* < 0.001). Tukey’s test showed a significant difference in MDA concentration between the pre-Aβ (*p* < 0.001) and post-Aβ (*p* < 0.001) groups compared to the control group. MDA concentration significantly decreased in the pre-treat group in comparison to the pre-Aβ group (*p* = 0.01, Figure 6A), and in the post-treat group in comparison to the post-Aβ group (*p* = 0.009, Figure 6A).

The plasma concentrations of TTG differ significantly among groups, as indicated by one-way ANOVA in pre-treat (F _(4, 35)_ = 11.43, *p* < 0.001) and post-treat (F _(4, 35)_ = 10.75, *p* < 0.001) groups. The TTG concentration was significantly lower in the pre-Aβ (*p* < 0.001) and post-Aβ (*p* < 0.001) groups than that in the control group. In addition, it was significantly increased after cacao treatment in the pre-treat group relative to the pre-Aβ group (*p* = 0.004, Figure 6B), and in the post-treat group relative to the post-Aβ group (*p* = 0.002, Figure 6B).

### 3.5 Congo red staining

**FIGURE 7:**
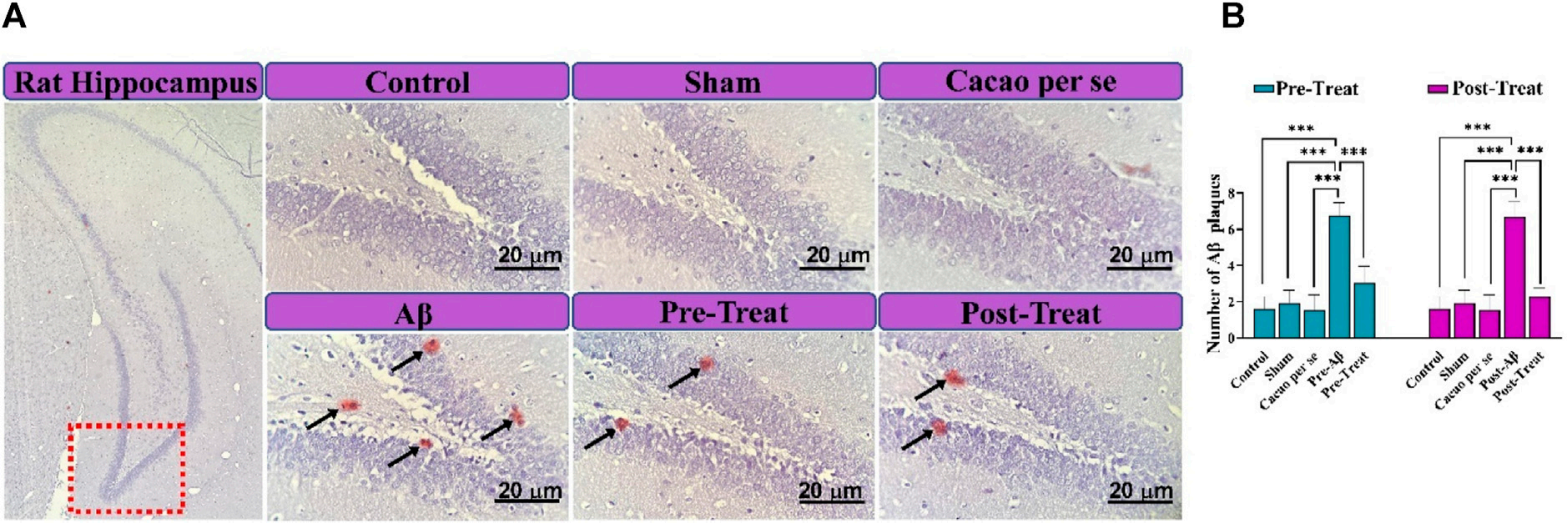
Congo red staining for review extracellular Aβ plaque deposition in the hippocampal DG region of rats. **(A)** Micrograph of extracellular Aβ plaques (Aβ plaques are seen in spots and marked with black arrows; scale bar = 20 μm). Fields were reviewed with a magnification of 400×. **(B)** Quantitative data of the number of Aβ plaques. Data are presented as means ± SD of four animals per group (one-way ANOVA and Tukey’s *post hoc* test). ***p < 0.001.

To confirm the formation of Aβ plaque in the rats’ brains, Congo red staining was conducted. As illustrated in Figure 7A, there was no noteworthy plaque in the control, Sham, and Cacao *per se* groups. The plaques found in the brain sections of the pre-Aβ (F _(4, 15)_ = 32.54, *p* < 0.001) and post-Aβ (F _(4, 15)_ = 38.65, *p* < 0.001) groups were significantly higher than those in control rats (*p* < 0.001). Interestingly, the amyloid plaque deposits were noticeably reduced in the pre-treat group in comparison to the pre-Aβ group (*p* < 0.001, Figure 7B), and in the post-treat group in comparison to the post-Aβ group (*p* < 0.001, Figure 7B).

## 4 Discussion

In the present study, passive avoidance memory was impaired in rats’ model of AD established by UICV injection of Aβ. This memory impairment was associated with an inhibition of LTP induction in the hippocampus’ PP-DG synapses, increased oxidative stress, and an increased accumulation of Aβ plaques in rats’ hippocampal DG. Conversely, treatment with cacao for two consecutive months mitigated passive avoidance dysfunction, ameliorated hippocampal LTP deficits, improved oxidant/antioxidant status, and inhibited Aβ plaque development in the Aβ-injected animals.

Chronic oral administration of cacao was investigated to see if it affected the cognitive memory associated with avoiding fearful contexts. In the passive avoidance test, rodents learn that entering the dark compartment is accompanied by an aversive stimulus (an electric foot shock). This indicates that rats’ cognitive abilities are reflected in their avoidance of entering the dark compartment. In the present study, Aβ-injection did not affect the acquisition phase. However, STLr was significantly reduced, whereas the TDC was enhanced in the AD rats compared to the control rats. These results suggest that Aβ-injection leads to impaired retention of passive avoidance memory in rats, which is in line with earlier studies (Gholipour et al., 2022; Samant and Gupta, 2022). Intriguingly, the STLr time was increased in the cacao-treated rats; hence, TDC time was decreased. The results showed that cacao could improve Aβ-associated passive avoidance memory deficiency. A similar finding has been reported in mice that supplementing their diet with LMN (containing cacao) improved their spatial cognition when aging and suffering from AD (Fernández-Fernández et al., 2012). Moreover, it was shown that chronic oral supplementation of Acticoa powder, a cacao polyphenolic extract, ameliorates age-related cognitive deficits in rats (Bisson et al., 2008). A clinical trial has revealed that daily consumption of cacao in old people with mild cognitive impairment showed considerable improvement in executive function and working memory (Desideri et al., 2012). However, in an animal model of AD, this is the first study to report that cacao was beneficial to passive avoidance memory deficits induced by Aβ injection.

To investigate the cellular basis of learning and memory in the hippocampus, LTP is used (Bliss and Collingridge, 1993). LTP can be induced through the activation of glutamate receptors of the N-methyl-D-aspartate (NMDA) type, which usually occurs during the simultaneous activation of presynaptic and postsynaptic neurons (Lüscher and Malenka, 2012). In the present study, Aβ injection affected synaptic function and inhibited hippocampal LTP induction in the DG region. The effects of Aβ injection on hippocampal synaptic plasticity were evident in both decreased PS amplitude and fEPSP slope. Previous evidence suggests that in the hippocampus, neuronal network dynamics and oscillations are impaired by Aβ application (Park et al., 2020; Andrade-Talavera and Rodríguez-Moreno, 2021). Soluble Aβ_1-__42_ oligomers can induce extensive neuronal loss in animal models and initiate a cascade of events that mimic several key aspects of AD (Brouillette et al., 2012).

There is considerable electrophysiological evidence from rodent models that Aβ injection can interfere with neuronal homeostasis, leading to LTP suppression of the hippocampal DG region and learning and memory deficits (Yang et al., 2021; Hu et al., 2022; Salimi et al., 2022). Evidence strongly suggests that Aβ increases presynaptic Ca^2+^ and alters glutamate levels at hippocampal synapses, leading to excitotoxicity (Tofighi et al., 2021). In addition, Aβ induces loss of cell-surface AMPA receptors using long-term depression (LTD) signaling pathways (Hampel et al., 2021). Aβ can also lead to the downregulation of NMDA receptors through endocytosis and changes in dendritic spine density (Vyas et al., 2020). The hippocampal synaptic function can be impaired as a result of these Aβ-induced detrimental alterations, followed by impairment of hippocampus-mediated learning and memory functions. In this regard, it has been demonstrated that passive avoidance memory decline induced by ICV administration of Aβ as a model of AD is closely related to the suppression of hippocampal LTP induction in the DG region (Aliakbari et al., 2021). However, treatment with cacao in the AD rats restored hippocampal LTP impairment by enhancing the fEPSP slopes and the PS amplitude after HFS for up to 60 min. The consumption of dark chocolate reverses the detrimental effects of chronic isolation stress on synaptic potency, hippocampal plasticity, and learning and memory in rats, which is in agreement with this study (Kalantarzadeh et al., 2023). According to the current study, cacao can improve the deteriorating effect of Aβ on DG-induced LTP in rats. As a result of this study, cacao may alleviate hippocampal LTP impairment in the granular cells’ layer of the DG, perhaps partially explaining cacao’s beneficial effects on Aβ-induced passive avoidance memory impairment.

The protective effects of cacao can be attributed, at least in part, to several mechanisms listed below: 1) enhancing cerebral blood flow (Brickman et al., 2014; Haskell-Ramsay et al., 2018), 2) potentiating neurotrophic factors (Sumiyoshi et al., 2019), 3) promoting neurogenesis in the subventricular zone and hippocampus (Fernández-Fernández et al., 2012), 4) improving the cholinergic neurotransmission in the hippocampus (Madhavadas et al., 2016), 5) modulating brain-derived neurotrophic factor (BDNF)/tropomyosin-related kinase B (TrkB) signaling pathway (Cimini et al., 2013), 6) activating nuclear factor-erythroid-2-related factor 2 (Nrf2)/heme oxygenase-1 (HO-1) pathway (Shah et al., 2010), 7) elevating cyclic adenosine monophosphate (cAMP)/cAMP-response element binding protein (CREB)/BDNF pathway (Yoneda et al., 2017), and 8) inhibiting mammalian target of rapamycin (mTOR) signal (Sugimoto et al., 2019).

Oxidative stress is regarded as the core pathogenesis of AD (Bai et al., 2022). It has been shown that antioxidant agents exert an ameliorative effect on the induction of hippocampal LTP, and subsequently improve AD-induced cognitive dysfunction (Ahmadi et al., 2021a; Nazifi et al., 2021). In the present study, Aβ administration showed an imbalance of oxidative–antioxidative status in the plasma of the rats, which was indicated by a decrease in the concentration of TTG (as a non-enzymatic antioxidant) and an increase in the MDA level (as an indicator of lipid peroxidation), which is in congruence with previous results (Komaki et al., 2019; Ahmadi et al., 2021b). TTG contributes to the greater part of the total antioxidants found in the body and plays an essential role in defense against ROS (Chianeh and Prabhu, 2014). The TTG concentration in plasma can serve as an indirect marker of antioxidant capacity (Taysi et al., 2002). A high concentration of MDA has been proposed as an important factor in the pathogenesis and neuronal damage of AD patients, which implies a direct relationship between its level and ROS production (Aybek et al., 2007; Distéfano et al., 2022). Intriguingly, treatment with cacao ameliorated plasma oxidative/antioxidative balance by elevating the TTG concentration and suppressing the augmentation of MDA level, representing its antioxidant capability. This might explain a portion of the antioxidative activity of cacao due to its effects on superoxide dismutase (SOD), an endogenous antioxidant, and total antioxidant capacity (TAC) (Ali et al., 2017). Consistent with these results, the antioxidant properties of cacao have been previously reported in different models of brain injury such as AD (Cimini et al., 2013), stroke (Singh et al., 2022), Parkinson’s (Coe et al., 2022), and diabetes (de Oliveira and Genovese, 2013). Moreover, it has been reported that after 8 weeks of supplementation with cacao powder, d-galactose-induced aging rat brains were found to have increased levels of free radical scavenging enzymes such as catalase and glutathione peroxidase (Yoo and Kim, 2021). It has been reported that after 5 weeks of oral administration, the combination of cacao with nutraceuticals moderates inflammation, antioxidant responses, GSK-3-Wnt/-catenin signaling, ER stress, and apoptosis in aluminum chloride-induced AD rats (Abu-Elfotuh et al., 2023). It may, therefore, be plausible to speculate that cacao’s ability to scavenge free radicals and prevent oxidative damage contributes to its protective effect against Aβ-induced hippocampal LTP impairment.

The extracellular presence of Aβ plaque in the brain is one of the key components of AD pathology (Rahman and Lendel, 2021). Neurons surrounding amyloid plaques display dystrophic neurites and synaptic loss in animal models of AD (Benilova et al., 2012). Consistent with previous studies (Karthick et al., 2019; Bazrgar et al., 2022), UICV injection of Aβ in rats caused an increase in Aβ plaque accumulation in the hippocampal DG region stained with Congo red. Interestingly, Aβ plaque formation was successfully inhibited by cacao treatment in the hippocampal DG of rats infused with Aβ. Evidence suggests that Aβ can excessively activate NMDA receptors in hippocampal neurons, resulting in an increase in Ca^2+^ levels and ROS production (De Felice et al., 2007). Furthermore, Aβ inhibits hippocampal LTP, which can be a consequence of excessive ROS production induced by Aβ (Ma et al., 2011). The accumulation of Aβ has been linked to cognitive decline in both human and animal AD models in previous studies (Näslund et al., 2000; Billings et al., 2005; Ferreira and Klein, 2011). Present evidence shows that cacao’s ability to inhibit Aβ plaque formation may be part of the explanation for its protective effect against Aβ-induced LTP impairment.

## 5 Conclusion

It is suggested that chronic cacao treatment ameliorates passive avoidance memory decline in AD rats by modulating oxidative status, improving hippocampal LTP impairment, and suppressing plaque accumulation in hippocampal DG. This study suggests that cacao may be a promising agent against AD-related cognitive decline. However, further research is required to evaluate the mechanisms behind the protective effect of cacao against AD-induced cognitive decline in detail, especially the mechanisms involved in its antioxidative properties.

## Data Availability

The original contributions presented in the study are included in the article/Supplementary Material; further inquiries can be directed to the corresponding author.
